# RNA-Seq Analyses of Midgut and Fat Body Tissues Reveal the Molecular Mechanism Underlying *Spodoptera litura* Resistance to Tomatine

**DOI:** 10.3389/fphys.2019.00008

**Published:** 2019-01-22

**Authors:** Qilin Li, Zhongxiang Sun, Qi Shi, Rumeng Wang, Cuicui Xu, Huanhuan Wang, Yuanyuan Song, Rensen Zeng

**Affiliations:** ^1^College of Crop Science, Fujian Agriculture and Forestry University, Fuzhou, China; ^2^State Key Laboratory of Ecological Pest Control for Fujian and Taiwan Crops, Fuzhou, China; ^3^College of Life Science, Fujian Agriculture and Forestry University, Fuzhou, China

**Keywords:** tomatine, *Spodoptera litura*, RNA-sequencing, *GSTS1*, RNAi

## Abstract

Plants produce secondary metabolites to provide chemical defense against herbivorous insects, whereas insects can induce the expression of detoxification metabolism-related unigenes in counter defense to plant xenobiotics. Tomatine is an important secondary metabolite in tomato (*Lycopersicon esculentum* L.) that can protect the plant from bacteria and insects. However, the mechanism underlying the adaptation of *Spodoptera litura*, a major tomato pest, to tomatine in tomato is largely unclear. In this study, we first found that the levels of tomatine in tomatoes subjected to *S. litura* treatment were significantly increased. Second, we confirmed the inhibitory effect of tomatine on *S. litura* by adding moderate amounts of commercial tomatine to an artificial diet. Then, we utilized RNA-Seq to compare the differentially expressed genes (DEGs) in the midgut and fat body tissues of *S. litura* exposed to an artificial diet supplemented with tomatine. In total, upon exposure to tomatine, 134 and 666 genes were upregulated in the *S. litura* midgut and fat body, respectively. These DEGs comprise a significant number of detoxification-related genes, including 7 P450 family genes, 8 glutathione S-transferases (GSTs) genes, 6 ABC transport enzyme genes, 9 UDP-glucosyltransferases genes and 3 carboxylesterases genes. Moreover, KEGG analysis demonstrated that the upregulated genes were enriched in xenobiotic metabolism by cytochrome P450s, ABC transporters and drug metabolism by other enzymes. Furthermore, as numerous GSTs were induced by tomatine in *S. litura*, we chose one gene, namely *GSTS1*, to confirm the detoxification function on tomatine. Expression profiling revealed that *GSTS1* transcripts were mainly expressed in larvae, and the levels were the highest in the midgut. Finally, when larvae were injected with double-stranded RNA specific to *GSTS1*, the transcript levels in the midgut and fat body decreased, and the negative effect of the plant xenobiotic tomatine on larval growth was magnified. These results preliminarily clarified the molecular mechanism underlying the resistance of *S. litura* to tomatine, establishing a foundation for subsequent pest control.

## Introduction

Plants produce toxic secondary metabolites that provide chemical defense against herbivorous insects and pathogens. For instance, plants can biosynthesize a variety of toxins and secondary metabolites, such as isoflavones, furanocoumarins, terpenoids, alkaloids and cyanogenic glycoside (Mithöfer and Boland, 2012; Medina et al., 2015). Isoflavonoids are a characteristic family of natural products in legumes known to mediate a range of plant-biotic interactions. In soybean (*Glycine max: Fabaceae*), multiple isoflavones are induced and accumulate in leaves after *Spodoptera litura* larvae attack (Nakata et al., 2016). Tomato (*Lycopersicon esculentum* L.), an economically important vegetable worldwide and a commonly used model plant for studying plant-insect interactions (Wei et al., 2011), can be attacked by many herbivorous insects, such as *Trialeurodes vaporariorum* (Westwood) and *Helicoverpa armigera* (Hubner), but tomato can biosynthesize chemical substances to defend against herbivorous insects. For example, tomatine (TOM), an important secondary metabolite found in high amounts in tomato, serves as a growth inhibitor of *Trypanosoma cruzi*, strain EP, in Liver Infusion Tryptose medium (Chataing et al., 1998). TOM can inhibit bacteria and herbivorous insects (such as *S. litura*) and is expected to be exploited as a biological insecticide.

The co-evolution of plants and their insect predators has resulted in remarkable development in insects, including the abilities to deter and detoxify host plant phytochemicals (Mithöfer and Boland, 2012). Insect detoxification enzymes typically include three main superfamilies: cytochrome P450 monooxygenases (P450s or CYPs for genes), glutathione S-transferases (GSTs) and carboxylesterases (CarEs) (Després et al., 2007). *S. litura* feeds on more than 290 species of plants belonging to 99 families and is one of the most destructive agricultural pests in tropical and subtropical regions worldwide (Zou et al., 2016). Numerous insect cytochrome P450 genes, which involved in detoxification of allelochemicals, have been identified by newly developed high-throughput sequencing technologies. For example, *CYP6AB14* in *S. litura* has been suggested to play a key role in detoxifying plant allelochemicals, such as xanthotoxin, coumarin and flavone (Wang et al., 2015). *CYP6B8* and *CYP321A1* in the generalist *Helicoverpa zea* have been shown to metabolize a variety of allelochemicals, such as flavone, *α*-naphthoflavone, chlorogenic acid, and indole-3-carbinol (Li et al., 2004; Rupasinghe et al., 2007). Insect GSTs also play important roles in detoxifying toxic compounds; for example, *GSTE1* in the midgut of *S. litura* may play an important role in the detoxification of chlorpyrifos, xanthotoxin and the heavy metal cadmium (Xu et al., 2015). In addition, *GSTE2* in *Anopheles gambiae* showed enzymatic activity to detoxify dichlorodiphenyltrichloroethane (DDT) (Ding et al., 2003). Meanwhile, CarEs are implicated in the metabolic resistance of many different classes of insecticides, and two CarE genes, *Pxae22* and *Pxae31* in *Plutella xylostella*, have been shown to be involved in fipronil resistance (Ren et al., 2015). Plant secondary metabolites have a detrimental and toxic effect on the growth and development of herbivorous insects. However, tomatoes remain vulnerable to many pests, such as *S. litura*, indicating that *S. litura* may have adapted to the tomato defense mechanism. Studies have reported that tomatoes with TOM are highly toxic to insect attack. However, the specific effects of TOM on *S. litura* and how *S. litura* adapts and metabolizes TOM has not yet been reported.

In insects, immune and detoxification systems respond quickly to chemical and biological stresses (Lemaitre and Hoffmann, 2007) and are well expressed in the midgut (Hao et al., 2003; Pauchet et al., 2009), suggesting that this organ is the site of exposure to many stressors. However, detoxification also takes place in the fat body and hemolymph (Enayati et al., 2005; Dubovskiy et al., 2011). Transcriptome sequencing can systematically recognize the transcriptional regulation of all genes in an organism. Prior to this study, RNA sequencing was used to investigate the honeybee response to biotic and abiotic environmental stressors by measuring the midgut transcriptional changes induced by the parasite *Nosema ceranae* and one neurotoxic insecticide (fipronil or imidacloprid) alone or in combination (Aufauvre et al., 2014). In addition, the transcriptomic profiles of midgut genes and Cry1Ac gene networks resulting from challenging *P. xylostella* with the Cry toxin have also been studied using RNA-Seq (Lei et al., 2014). In addition, RNA-Seq and molecular docking reveal that *CYP397A1V2* likely contributes to P450-mediated insecticide resistance in *Cimex lectularius* (Mamidala et al., 2012). Understanding the effects of plant secondary metabolites on the feeding behavior, growth and development of insects and clarifying the mechanisms by which insects metabolize and adapt to plant secondary metabolites is very significant for pest management practices. However, the transcriptional levels of *S. litura* midgut and fat body genes induced by TOM have not yet been reported.

In this study, we used RNA-Seq analyses of the *S. litura* midgut and fat body to reveal the molecular mechanism underlying *S. litura* resistance to TOM. First, we observed that the TOM content in tomatoes subjected to *S. litura* attack was significantly increased. Second, to verify the inhibitory effect of TOM on *S. litura*, we fed *S. litura* artificial diets supplemented with moderate commercial TOM. Then, we used RNA-Seq to analyze the TOM-induced detoxification enzyme genes in the midgut and fat body tissues of *S. litura*. The differentially expressed genes (DEGs) and their associated pathways identified provide insight into the genes adapted to metabolize TOM. Finally, the *GSTS1* gene was silenced with RNA interference (RNAi) to further determine the likely contribution of *GSTS1*-mediated TOM resistance in *S. litura*.

## Materials and Methods

### Insect Culture, Plants and Antibodies

*Spodoptera litura* was provided by the Institute of Crop Resistance & Chemical Ecology of Fujian Agriculture and Forestry University and maintained in an insectary without exposure to any insecticide. Larvae were reared on artificial diets composed of soybean powder (100 g), brewer’s yeast (40 g), wheat bran (60 g), ascorbic acid (4 g), methyl p-hydroxybenzoate (2 g), sorbic acid (2 g), agar (16 g), cholesterol (0.8 g) and water (1 L) (Qi et al., 2000) at 25 ± 2°C and 70 ± 5% relative humidity with a photoperiod of 16:8 h (L:D). Adults were provided supplemented with 10% honey solution under the same conditions (Zhou et al., 2012).

Tomato (*L. esculentum* L.) cv Castlemart (CM) was used as the wild-type species for all experiments (Yan et al., 2013), and all tomato seeds were provided by Prof. Chuan-you Li (Genetics and Developmental Biology, Chinese Academy of Sciences, Beijing, China). Tomato seedlings were grown in growth chambers and maintained under 16 h of light (Yan et al., 2013) at 22°C and 8 h of darkness at 18°C and 60% relative humidity. Five-week-old plants with five to seven leaves were used in the experiment.

Tomatine (TOM) (90% purity, T0329, Tokyo Chemical Industry CO. LTD, Japan) was dissolved in dimethyl sulfoxide (DMSO, Q/STXH234-2013, XiLong Chemical Industry, Guangdong, China) and mixed with an artificial diet. The control diets were supplemented with the same amount of DMSO.

### Determination of Tomatine in CM Tomato Using HPLC

To examine the effects of *S. litura* damage on the changes of TOM content in wild-type tomatoes, we placed three fourth instar *S. litura* larvae on the fully expanded leaves of each 5-week-old tomato plant for 24 h. Control plants were not infested by *S. litura* larvae. After 24 h inoculation, tomato leaves from *S. litura*-infested plants and uninfested plants were weighed for TOM extraction. The TOM extraction method was referenced previously with some modifications (Kozukue et al., 2004). Briefly, 1. 200 mg tomato leaves were extract with 100 mL Chloroform/Methanol (2:1, v/v); 2. Add 2 mL of 0.2 N Hydrochloric acid; 3. Add 3 mL of 2% Ammonia, centrifuge at 18,100 × *g*/min for 1 min at 1°C, discard the supernatant and repeat the previous step; 4. Dissolve the precipitate with 2 mL of Tetrahydrofuran/Acetonitrile/0.02 M Monobasic potassium phosphate (50:30:20, v/v/v), centrifuge at 18,100 × *g*/min for 1 min at 1°C; 6. Pipette 1 mL into the sample vial for HPLC analysis.

To determine the sample TOM contents, HPLC analysis was carried out using a Waters liquid chromatography (model e2695), and clear supernatant extracts were injected into a stainless steel HPLC column (250 mm × 4.0 mm) filled with Inertsil ODS-2 (5 μM particles) and eluted with a mobile phase comprising acetonitrile/20 mM KH_2_PO_4_ (24:76) at a flow rate of 1 mL/min^-1^. The UV detector (model 2998 PDA) was set at 208 nm. The standard substance of tomatine (≥97%, HPLC, Chengdu Purechem-Standard CO. LTD, China) were used to confirm and quantify the peaks from TOM extraction.

### Insect Treatment, Sample Collection and RNA Extraction

Newly molted fourth instar *S. litura* larvae were used for all treatments to monitor weight growth rate. Synchronous larvae (80–100 mg) were first weighed (labeled as W_T1_) and fed artificial diets supplemented with TOM at 0.1 and 0.3 mg/g for 48 h. The control larvae were fed on artificial diet supplemented with the same amount of DMSO (labeled as W_C1_). The weight of larvae were measured again simultaneously after inoculation (for TOM treatment group, W_T2_; for control group, W_C2_). The relative weight growth rate of larvae from treatment group were calculated by normalized with control larvae, that is, (W_T2_-W_T1_)/(W_C2_-W_C1_) × 100%. Thirty synchronous individuals were used for each treatment, and three independent replicates were performed for all treatments.

After 48 h inoculation, midgut and fat body tissues from three *S. litura* larvae were, respectively, dissected prior to RNA extraction. Each treatment had four replicates. Total RNA was isolated from flash-frozen tissues using the Eastep Super Total RNA Extraction Kit (Promega Corporation, Madison, WI, United States) and quantified by measuring the absorbance at 280 and 260 nm. Then the equal RNA from four replicates were pooled together as a mix sample, including midgut and fat body tissues from control or TOM-treated samples. The pooled samples were subjected to RNA-Seq, and the four replicates samples were used for qRT-PCR analysis to verify the results of RNA-Seq and the induced effect of TOM stress.

### Library Preparation and Sequencing

Total RNA was quantified by the NanoPhotometer^®^ spectrophotometer (IMPLEN, United States) and RNA quality was assessed using the RNA Nano 6000 Assay Kit in the Bioanalyzer 2100 system (Agilent Technologies, United States). The transcriptome libraries were generated using Illumina TruSeq^TM^ RNA Sample Preparation Kit (Illumina, San Diego, CA, United States) following the manufacturer’s recommendations. RNA transcript was sequenced on an Illumina Hiseq 2000 in Novogene Bioinformatics Institute (Beijing, China).

### Quantification and Differential Expression Analysis of Transcripts

Raw data (raw reads) in FASTQ format were processed through in-house Perl scripts to remove reads containing adapters, reads containing ploy-N, and low quality reads. Q20, Q30 and GC-content of the cleaned data were used to assess the sequencing quality. All the downstream analyses were based on clean data with high quality. A global *de novo* assembly of the resultant reads was performed using the Trinity method with min_kmer_cov set to 2 by default and all other parameters set default. To annotate the obtained unigenes, the databases of Nr (*e*-value ≤ 1*e*-5), Nt (*e*-value ≤ 1*e*-5), Pfam (*e*-value ≤ 1*e*-2), KOG/COG (Supplementary Figures 2, 3) (*e*-value ≤ 1*e*-3), Swiss-protc (*e*-value ≤ 1*e*-5), KEGG (*e*-value ≤ 1*e*-10), and GO (*e*-value ≤ 1*e*-6) were searched.

For reads mapping, the transcriptome obtained by Trinity splicing was used as reference sequence, and all clean reads were mapped to the reference sequence using RSEM with bowtie 2 set to mismatch 0 by default (Li and Dewey, 2011). Splicing length and frequency distribution of transcripts and unigenes were listed in Supplementary Table 4 and success rate of gene annotation were listed in Supplementary Table 5. For quantification of gene expression level, the number of expressed reads mapped to each gene was calculated and normalized to the number of FPKM (expected number of Fragments Per Kilobase of transcript sequence per Millions base pairs sequenced) (Trapnell et al., 2010).

The read counts were normalized using the edgeR Bioconductor (Robinson et al., 2010) with the TMM method (Storey, 2003), and the DESeq R package provided statistical routines for determining differential expression using a model based on the negative binomial distribution, and was used to identify DEGs between the control and TOM-treated samples. The *p*-values in multiple tests were adjusted as *q*-values using the Benjamini and Hochberg’s approach for controlling the false discovery rate (FDR) (Dillies et al., 2013). We used “fold changes ≥ 1 and *q* < 0.005” as the threshold to assess DEGs between the TOM treatment and control groups.

### Quantitative Real-Time PCR (qRT-PCR) Analysis

To validate the DEGs analysis results, quantitative real-time reverse transcriptase PCR (qRT-PCR) experiments were performed on an Applied Biosystems StepOne Plus Real-Time PCR System in a 10 μL reaction volume consisting of 5 μL of 2× SYBR GoTaq^®^ qPCR Master Mix (Promega Corporation, Madison, WI, United States), 0.4 μL of each gene-specific primers (10 μM), 1 μL cDNA equivalent to 50 ng total RNA and sterilized water to reach the final volume. PCR conditions were set as: 1 cycle of 95°C for 10 min; 40 cycles of 95°C for 15 s, 55°C for 30 s and 72°C for 30 s. The reference gene elongation factor 1 alpha (EF-1α) was used as internal controls (Shu et al., 2018). A dissociation curve analysis program was performed to check the homogeneity of the PCR product. Relative standard curves of EF-1α and target genes were generated by using 10-fold serial dilutions cDNA to calculate the amplification efficiency of primers. The relative mRNA levels were normalized against EF-1α using the 2^−Δ Δ *Ct*^ method (Livak and Schmittgen, 2001). Three independent biological repeats were performed, each sample had two technical replicates, and a calibrator sample was used to make comparisons between different plates. All the primers were listed on Supplementary Table S1. All designed primers were synthesized at BioSune Biotechnology Co., Ltd. (Shanghai, China).

### Clone of the *GSTS1* Gene

To develop full-length *GSTS1*, we performed 3′ RACE and 5′ RACE (rapid amplification of cDNA ends) using an oligo dT primer (Invitrogen) and gene-specific primers. The following gene-specific primers were utilized: 5′ RACE primer 1, 5′-GCCATCACCAAGTATGTGGCAAGAGGA-3′; 5′ RACE primer 2, 5′-TGGGGTGATTGAAGCCAGCGACAT-3′; 3′ RACE primer 1, 5′-GATAATCCTTGCTCAATTCGATGCCCAG-3′ and 3′ RACE primer 2, 5′-CACCCCAGGAAAGCTTGCCATTCA-3′. By merging the 3′ and 5′ cDNA ends with internal fragment sequences, full-length cDNAs of *GSTS1* were generated and then deposited into the GenBank database (accession number: KY304480.1^1^).

### Preparation and Injection of dsRNA

Templates for *in vitro* transcription reactions were prepared by PCR amplification using cloned *GSTS1* sequences as the template and the primers (T7-*GSTS1*dsRNA-F/*GSTS1*dsRNA-R and *GSTS1*dsRNA-F/T7-*GSTS1*dsRNA-R). The amplification conditions comprised 30 cycles at 98°C for 10 s, 53°C for 30 s and 72°C for 45 s, with a final extension step at 72°C for 5 min. PCR products were purified using the TIANGEN Universal DNA purification kit (Tiangen Biotech, Co., Ltd., Beijing, China), and DNA concentrations were determined using a microplate reader. Double-stranded RNA (dsRNA) corresponding to *GSTS1* (ds*GSTS1*) was synthesized using the T7 RiboMAX^TM^ Express RNAi System (Promega, United States) according to the manufacturer’s instructions. Additionally, 688-bp dsRNA corresponding to the control green fluorescent protein (GFP) gene (ACY56286), used as a negative control, was synthesized by the same method using the following primer (T7-*GSTS1*dsRNA-F/*GSTS1*dsRNA-R and *GSTS1*dsRNA-F/T7-*GSTS1*dsRNA-R) pairs: T7-GFPdsRNA-F/GFPdsRNA-R and GFPdsRNA-F/T7-GFPdsRNA-R (Dong et al., 2011). The resulting dsRNA were analyzed by agarose gel electrophoresis and stored at -80°C prior to use. All the primers were listed on Supplementary Table S3.

The dsRNA were adjusted to a final concentration of 1.5 μg/μL with ddH_2_O prior to use. For all dsRNA injection experiments, fourth instar larvae were used; 2 μL (3.0 μg) of dsRNA was injected into the side of each *S. litura* thorax using a manual microliter syringe (Shanghai High Pigeon Industry and Trade Co., Ltd., China), and the injection points were imprinted immediately with Vaseline. Following injection, *S. litura* were maintained on artificial diets supplemented with or without 0.1 mg/g TOM. The treatment larvae were injected with 3.0 μg ds*GSTS1*, while the control larvae were injected with equal ds*GFP*. At 24 h post injection, insect midguts and fat bodies were harvested, and total RNA were extracted as described above.

### Statistical Analysis

All data are presented as the mean ± SE unless otherwise noted. Statistically significant differences (*p* < 0.05) were determined by one-way ANOVA followed by Duncan’s multiple range test using the SPSS 10.0 software package (IBM Corp., Armonk, NY, United States).

## Results

### Tomatine Contributes to Tomato Chemoresistance Against *S. litura*

**FIGURE 1:**
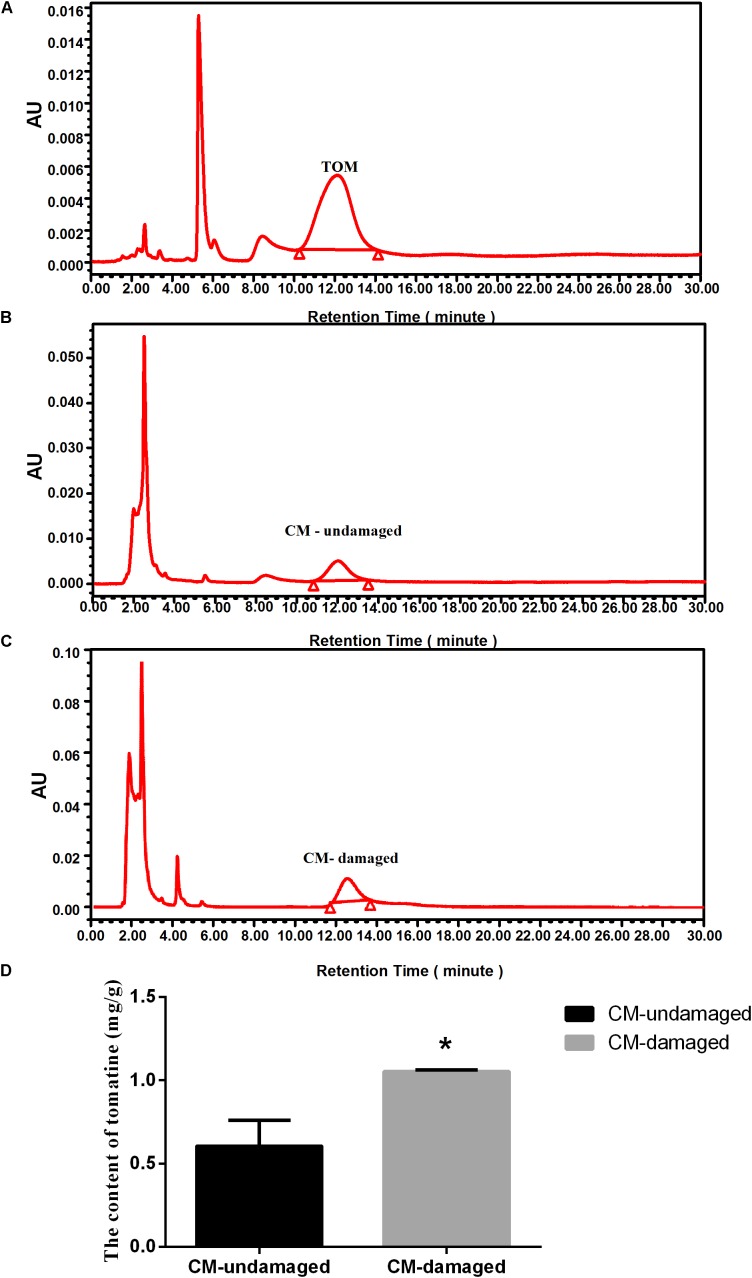
Effect of *S. litura*-damage on tomatine content in CM tomatoes. **(A)** HPLC chromatographic profiles of the standard substance of TOM. **(B)** HPLC chromatographic profiles of TOM extracted from wild-type undamaged tomatoes. **(C)** HPLC chromatographic profiles of TOM extracted from *S. litura*-damaged CM tomatoes. **(D)** Contents of TOM in wild-type plants before and after *S. litura* damage. The tomatine concentration is expressed as mg/g solution for HPLC detection (*n* = 5, ^∗^*p* < 0.05, *t*-test).

We hypothesized that tomatoes might produce tomatine (TOM), an important toxic compound, to improve resistance to insect damage. To test this hypothesis, we used HPLC to determine the TOM content in *S. litura* damaged wild-type tomatoes (cv Castlemart, CM) and *S. litura* undamaged CM. Compared with standard substance of TOM (Figure 1A), the peaks of TOM extracted from CM-undamaged (Figure 1B) and CM-damaged (Figure 1C) were confirmed. The physiological levels of TOM in undamaged tomatoes were 0.61 ± 0.15 mg/g, however, when damaged by *S. litura*, the TOM content were significantly increased to 1.05 ± 0.01 mg/g (Figure 1D). The results verified our hypothesis that TOM contributes to tomato’s defense against *S. litura* attack.

### The Effect of Exposing to Tomatine on Growth of *S. litura* and Morphological of Midgut Tissue

**FIGURE 2:**
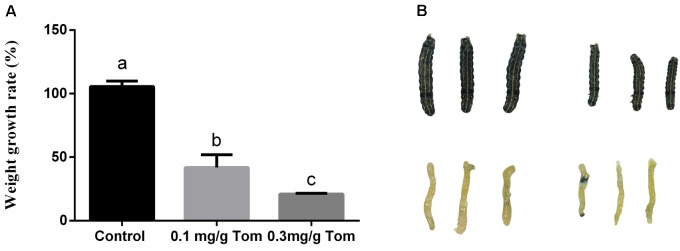
The effect of exposing to tomatine on growth of *S. litura* and morphological of midgut tissue. **(A)** Effects of the presence and absence of tomatine in artificial diets (TOM, 0.1 mg/g, 0.3 mg/g) on weight growth rate (*n* = 10, *p* < 0.05, Dunnett’s multiple range test). **(B)** Morphological changes in the phenotype and midguts of larvae fed a diet supplemented with tomatine; Control: larvae fed a diet supplemented with DMSO; tomatine: larvae fed a diet supplemented with 0.1 mg/g tomatine.

Next, biological experiments were performed on fourth instar *S. litura* larvae maintained on artificial diets supplemented with pure TOM. In comparison with control larvae reared in the absence of TOM, the average weight gains observed for fourth instars fed diets containing 0.1 mg/g TOM were decreased by 54% following 48 h of treatment (Figure 2A). And *S. litura* fed artificial diets supplemented with 0.3 mg/g pure TOM were decreased by 80% (Figure 2A). Then we confirmed the growth inhibition phenotype of *S. litura* induced by TOM, revealing that larvae treated with TOM were significantly smaller (Figure 2B). To determine the effect of TOM on insect histomorphology, midgut samples of larvae were dissected and measured, revealing that the larval midgut was significantly smaller after TOM exposure (Figure 2B).

### Analysis of DEGs in the Midgut and Fat Body Tissues of *S. litura* After TOM Treatment

**FIGURE 3:**
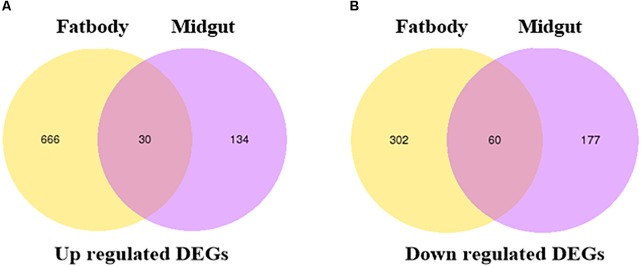
Venn diagram illustrating the differentially expressed genes. **(A)** Venn diagram of upregulated DEGs in the fat bodies and midguts of *S. litura* subjected to tomatine treatment. **(B)** Venn diagram of downregulated DEGs in the fat bodies and midguts of *S. litura* subjected to tomatine treatment. The *q*-values in multiple tests were adjusted to control the false discovery rate (FDR). We used “fold changes ≥ 1 and *q* < 0.005” as the threshold to assess differentially expressed genes between the TOM treatment and control groups.

To study the molecular mechanism underlying the *S. litura* counterdefense against TOM, we utilize RNA-Seq to explore differences in gene expression in midgut and fat body tissues after TOM treatment. *S. litura* larvae were maintained on artificial diets supplemented with TOM for 24 h. We then dissected the midgut and fat body tissues of *S. litura* and analyzed the DEGs by RNA-Seq. In total, 134 upregulated genes and 177 downregulated genes were observed in the midgut, whereas 666 upregulated genes and 302 downregulated genes were observed in fat body tissue (Figure 3, fold changes ≥ 1, *q* < 0.005). We mainly focused on screening detoxification genes; gene family enrichment analysis was performed on DEGs identified in each group, including P450 family genes, ABC transport enzymes, UDP-glucosyltransferases, CarEs, and glutathione transferases (Table 1). We focused mostly on upregulated genes, including 2 P450 family genes, 3 ABC transport enzymes, 5 UDP-glucosyltransferases and 2 CarEs in the midgut and 5 P450 family genes, 1 CarE, 8 glutathione transferases, 3 ABC transport enzymes and 4 UDP-glucosyltransferases in fat body tissues (Table 1).

**Table 1:** Up-regulated differentially expressed genes in Midgut and Fat body.

Gene Family	Genes	Length	NR description	Tissue
CYPs	c37793_g1	2044	Cytochrome P450 CYP4L4	M
	c41002_g1	2741	Cytochrome P450	M
	c20728_g1	3182	Cytochrome CYP4G75	F
	c246_g1	2553	Cytochrome CYP324A6, partial	F
	c38103_g2	2881	Cytochrome CYP340AB1	F
	c40402_g10	2389	Cytochrome P450	F
	c82532_g1	3240	Cytochrome P450 CYP339A1	F
ABC	c29620_g1	3218	ATP-binding cassette sub-family G member 1	M
	c40177_g2	5992	Probable multidrug resistance-associated protein lethal(2)03659	M
	c93246_g1	3848	ATP-binding cassette sub-family G member 4-like	M
	c28026_g1	3967	ABC transporter F family member 4-like	F
	c30414_g1	5686	ATP-binding cassette sub-family A member 2-like	F
	c39649_g1	5573	ATP-binding cassette transporter subfamily B isoform X1	F
UGTs	c39571_g1	1616	UDP-glycosyltransferase 33T2	M
	c39987_g1	1962	UDP-glycosyltransferase 40U1	M
	c40154_g1	2886	UDP-glycosyltransferase UGT40Q1	M
	c64266_g1	786	UDP-glucuronosyltransferase 2B18-like	M
	c92438_g1	1444	UDP-glucose 4-epimerase-like	M
	c20995_g1	2726	UDP-glycosyltransferase UGT42C1	F
	c39551_g1	1660	UDP-glycosyltransferase 33J2	F
	c39991_g8	2294	UDP-glycosyltransferase 33F4	F
	c39991_g9	1988	UDP-glycosyltransferase 33B13	F
CCEOs	c40774_g1	1903	Carboxyl/choline esterase CCE016a	M
	c82003_g1	1894	Carboxyl/choline esterase CCE025a	M
	c31996_g2	3159	Carboxyl/choline esterase CCE006a	F
GSTs	c21945_g1	685	Glutathione S-transferase s3 protein	F
	c27402_g1	1389	Glutathione S-transferase epsilon 11	F
	c27782_g2	832	Glutathione S-transferase epsilon 2	F
	c36666_g1	1007	Glutathione S-transferase epsilon 13, partial	F
	c82114_g1	1278	Glutathione S-transferase s2 protein	F
	c92462_g1	748	Glutathione S-transferase GSTS1	F
	c92688_g1	1051	Glutathione S-transferase zeta 2	F
	c72041_g1	898	Glutathione S-transferase sigma 5	F


*M, midgut; F, fat body*.

KEGG pathway enrichment analysis was done using KOBAS 2.0 with a hypergeometric test and the Benjamini-Hochberg FDR correction (Xie et al., 2011). KEGG analysis demonstrated that the upregulated genes were enriched in xenobiotic metabolism by cytochrome P450, ABC transporters, and drug metabolism by other enzymes (Table 2). These results showed that numerous detoxification enzymes were induced in *S. litura* subjected to TOM treatment.

**Table 2:** KEGG pathways containing genes differentially expressed in Midgut and Fat body.

KEGG term	*p* Value	Tissue
ko02010: ABC transporters	0.0061	M
ko00983: drug metabolism—other enzymes	0.0501	M
ko00980: metabolism of xenobiotics by cytochrome P450	0.0178	F
ko00480: glutathione metabolism	0.0085	F


*M, midgut; F, fat body*.

### Verification of RNA-Seq Results by qRT-PCR

**FIGURE 4:**
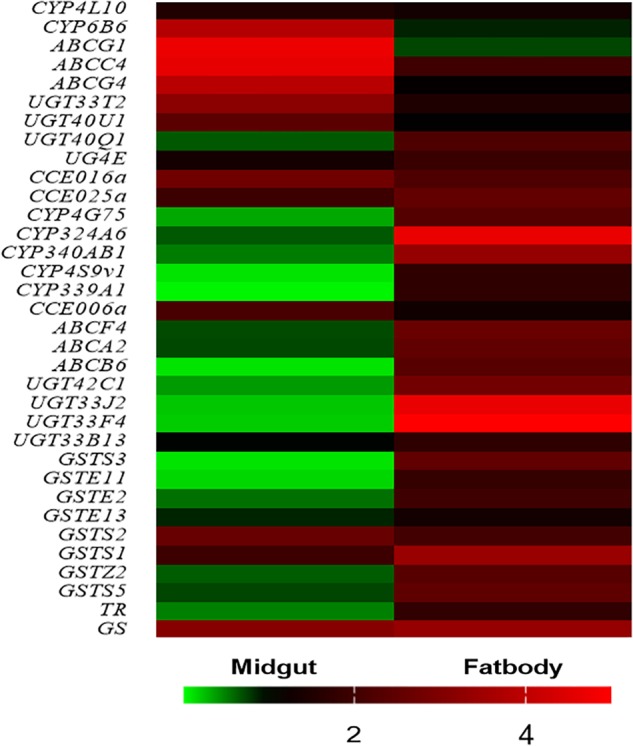
Biological (independent) qRT-PCR validation of the RNA-Seq data. The first 11 genes are highly expressed in the midgut when exposure to tomatine (TOM), and the latter 23 genes are highly expressed in fat bodies when exposure to TOM. The bar represents the scale of the expression levels of TOM treatment/Control. The red, black, and green bar indicates mRNA expression levels of TOM treatment are higher, similar or lower than the control groups, respectively. The detailed fold change and *p* value were shown in Supplementary Table S2.

To validate the gene expression data obtained using RNA-Seq, we combined 30 genes which were commonly upregulated in midgut and fat body tissues (Figure 3A) with the reported genes and KEGG analysis, and 34 DEGs were selected for quantitative real-time PCR (qRT-PCR) analysis. The heat map showed that almost all the upregulated genes of in midgut and fat body from RNA-seq data were also upregulated in the qRT-PCR analysis in biological replicates samples, except for the *UGT40Q1* gene in midgut (Figure 4), which was upregulated in TOM treatment from RNA-seq, but was downregulated in the qRT-PCR analysis (Supplementary Table S2). The detailed fold change and *p* value were shown in Supplementary Table S2. The 97.06% (33/34) consistency of gene expression indicated that the RNA-Seq approach provided reliable differential gene expression data for this system.

### Sequence Analysis and Spatial and Temporal Expression of *GSTS1*

Transcriptome sequencing data revealed that multiple GST family genes were induced by TOM in *S. litura*, suggesting that GST family genes may play an important role in the counterdefense of *S. litura* to TOM. In addition, we identified a new GST family gene, whose function has not yet been reported, *GSTS1*, which we utilized for further functional studies. Using RACE, the full-length 748-bp *GSTS1* cDNA sequence was determined to contain a 36-bp 5’-untranslated region (5’-UTR), a 639-bp open reading frame (ORF), and a 78-bp 3’ UTR. The sequence was deposited into the GenBank database (accession number: KY304480.1). The ORF encodes a predicted protein of 213 amino acids. *GSTS1* has a theoretical pI value of 6.62 and a predicted molecular mass of 24.57 kDa.

**FIGURE 5:**
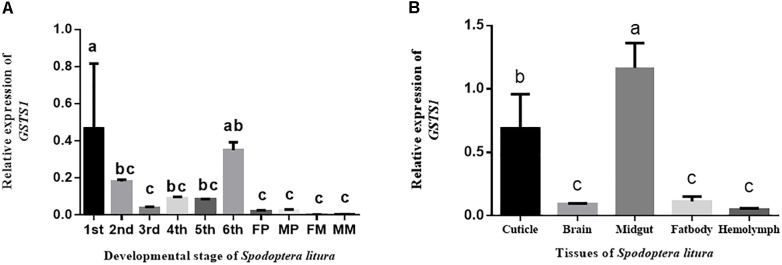
The qRT-PCR analysis of *GSTS1* expression in various *S. litura* tissues and life stages. **(A)** Relative spatial expression of *GSTS1*; the different life stages include 2-day-old 1st, 2nd, 3rd, 4th, 5th, and 6th (last) instar larvae, pupae, and adults. **(B)** Relative temporal expression of *GSTS1*; the different tissues include the cuticle, brain, midgut, fat body, and hemolymph. The different letters on the error bars indicate significant differences (*p* < 0.05, Duncan’s multiple range test) among the developmental stages and tissues.

To study the spatial expression of *GSTS1* in *S. litura*, we use RT-qPCR to test the relative expression patterns of *GSTS1* mRNA at different ages. *GSTS1* transcripts were mainly present in larvae, especially in first instar larvae, and slightly lower transcript levels were observed in female moths (Figure 5A). To study the temporal expression of *GSTS1* in *S. litura*, RT-qPCR was used to test the relative expression patterns of *GSTS1* mRNA at different developmental stages. Among the stages analyzed, *Slitu*-*GSTS1* was almost exclusively expressed in the midgut (Figure 5B). The expression of *GSTS1* was negligible in brain, fat body and hemolymph tissue. Thus, we speculated that the high *GSTS1* expression in the midgut is likely related to metabolizing TOM in this region.

### Effect of Silencing *GSTS1*

**FIGURE 6:**
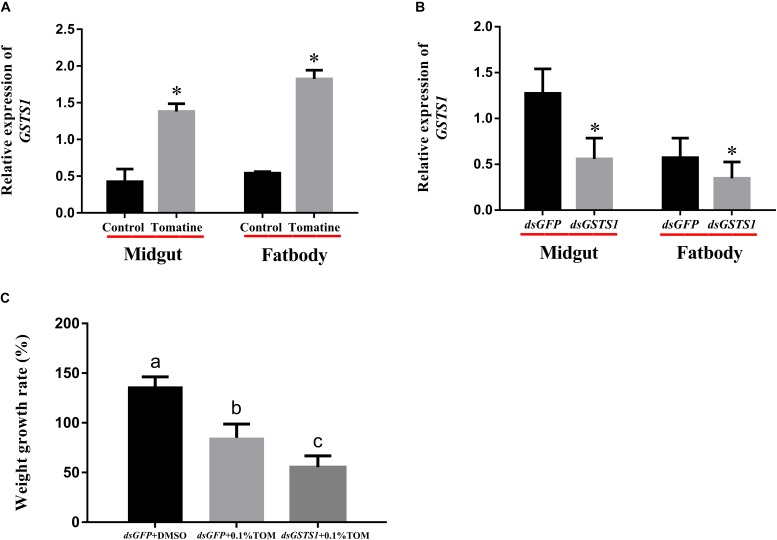
Effect of dsRNA injection on endogenous *GSTS1* transcript levels. **(A)** The effects of TOM treatment on *GSTS1* transcript levels were determined by quantitative real-time RT-PCR. **(B)** Steady-state *GSTS1* transcript levels in midgut and fat body tissues were determined by the quantitative real-time RT-PCR analysis of green fluorescent protein dsRNA (*dsGFP*) and *GSTS1* dsRNA (ds*GSTS1*) injections into fourth instar *S. litura* larvae. **(C)** Effects of dsRNA injection on the weight gain and mortality of *S. litura* larvae fed TOM. The weight growth rates of *S. litura* fourth instar larvae fed artificial diets supplemented with tomatine (TOM, 0.05 mg/g) after receiving dsRNA injections at different feed stages were monitored. Data were normalized to the α-actin internal control, and the 2^−Δ Δ *Ct*^ method was used to determine the relative expression levels. The results are expressed as the mean ± SE from assays performed in triplicate. Asterisks indicate significant differences (^∗^*p* < 0.05). Significant differences among groups are indicated by the letters above each bar (*p* < 0.05 according to one-way ANOVA followed by the Duncan’s multiple range test). *dsGFP*+TOM, green fluorescent protein dsRNA-injected larvae fed artificial diets supplemented with tomatine; ds*GSTS1*+TOM, *GSTS1*-injected larvae fed artificial diets supplemented with tomatine.

We validated the *GSTS1* gene expression data by qPCR, revealing that *GSTS1* was transcribed in both the midgut and fat body (Figure 6A). From the above results, we hypothesized that *GSTS1* mediated the resistance of *S. litura* to TOM. To further confirm this function, the *GSTS1* gene was knocked down by injecting dsRNA into fourth instar larvae. Approximately 24 h after the injection, RT-qPCR showed that *GSTS1* expression was significantly reduced in the midguts and fat bodies of fourth instar *S. litura* larvae subjected to the dsRNA injection compared to that in control larvae (received a double-stranded GFP, *dsGFP*) injection (Figure 6B), showing that the RNAi procedure was successful.

RNAi experiments were next performed on fourth instar larvae maintained on artificial diets supplemented with TOM. Importantly, larvae injected with *dsGSTS1* and fed TOM exhibited significantly lower weight gains than *dsGFP*-injected controls fed TOM (Figure 6C), showing that *GSTS1* may plays an important role in metabolizing TOM.

## Discussion

Insect organs, especially the midgut and fat body, are important for defense against xenobiotic compound toxicity. A previous study reported the transcription and expression of genes in the midgut tissue of *Bombyx mori* strain Daizo larvae subjected to persistent pathogenic infection with cytoplasmic polyhedrosis virus (BmCPV) (Kolliopoulou et al., 2015). Another study determined that heat shock proteins (HSPs) and their expression levels may play important roles in the resistance of various silkworms to high temperature stress by analyzing gene expression in their midguts (Li et al., 2014). In addition, another study reported the physiological shift of pre-blood-fed fat bodies from a resting state to vitellogenic gene expression after conducting transcriptome analysis of fat bodies of the yellow fever mosquito *Aedes aegypti* (Price et al., 2011). Prior research has suggested that the *S. litura* midgut plays a crucial role in growth physiology by influencing digestion and metabolism (Franzetti et al., 2015). In our study, we performed RNA-Seq analysis on midgut and fat body tissues to investigate the mechanisms underlying tomatine (TOM) resistance. In total, 134 and 666 upregulated genes were identified in *S. litura* midgut and fat body tissues, respectively, among which 30 genes were commonly differentially expressed. These results suggested that these genes may play a substantial role in TOM detoxification.

We classified DEGs according to their gene family and compared the differences in gene expression between midgut and fat body tissues. The results suggested that GSTs were significantly differentially expressed between control *S. litura* and *S. litura* treated with TOM. RNAi was performed on *GSTS1* to further reveal the molecular mechanism underlying the resistance of *S. litura* to TOM. Previous studies on metabolic resistance have focused on the roles of P450s, CarEs, and GSTs in xenobiotic metabolism in the Lepidoptera midgut (Pauchet et al., 2010). Current research on GSTs has focused mainly on the relationship between GSTs and insecticide resistance in insects. For example, the organophosphorus insecticide chlorpyrifos increased the amounts of both GSTs and malonaldehyde in *S. litura* (Huang et al., 2011). *GSTe2* and *GSTe7* of *A. aegypti* are involved in resistance to pyrethroid deltamethrin (Lumjuan et al., 2011). In *S. litura*, both *SlGSTe2* and *SlGSTe3* responded to six insecticides, but *SlGSTE2* showed a much higher detoxification activity than *SlGSTE3* (Deng et al., 2009). Moreover, different GSTs in a species have different detoxification activities against various toxic compounds. Eight GSTs have been identified in *S. litura*, and the mRNA levels of *SlGSTe1, SlGSTe3, SlGSTs1, SlGSTs3* and *SlGSTo1* were shown to be increased after xanthotoxin ingestion (Huang et al., 2011). *SlGSTE1* was shown to play a potentially critical role in *S. litura* host adaptation (Zou et al., 2016). However, plant allelochemicals can also present their toxicity via the oxidative stress pathway. For example, xanthotoxin, a plant allelochemical from *Apiaceae*, can generate superoxide anion radicals, hydrogen peroxide and hydroxyl radicals, causing deleterious lipid peroxidation and increasing the antioxidative activity of glutathione peroxidase in several insects (Ahmad and Pardini, 1990). TOM is a tetrasaccharide linked to the 3-OH group of the aglycone tomatidine (Supplementary Figure S1) and could induces permeabilisation of the cell membrane and a loss of the cytosolic enzyme pyruvate kinase (Medina et al., 2015). Intriguingly, as some environmental compounds induce excessive GST expression, certain GSTs have been utilized as biomarkers of environmental pollution (Pérez-López et al., 2002; Ayoola et al., 2011), and GSTs have many other functions that remain to be explored. In this study, we further studied the contribution of the *GSTS1* gene to mediating TOM resistance in *S. litura*, as this relationship has not yet been reported.

In our study, we suggest that *GSTS1* expression is increased to metabolize TOM, thus revealing the molecular mechanism by which *S. litura* mediates TOM resistance. Furthermore, in addition to *GSTS1*, many other genes, such as P450s, CarEs, glutathione transferases, ABC transport enzymes and UDP-glucosyltransferases, may also mediate TOM resistance in *S. litura*. For example, a member of the CYP6 family, CYP6A8, catalyzes the hydroxylation of lauric acid and increase the resistance of Drosophila melanogaster against aldrin and heptachlor (Restifo, 2004). Many important molecules, including pheromones and other semiochemicals, are types of esters that are hydrolysed by esterases in insects (Montella et al., 2012). Further research should establish whether these genes function individually or in combination to mediate resistance, and their functions still need to be verified.

RNAi has been developed as an effective tool in plants and animals (Plasterk et al., 2000; Aravin et al., 2001; Wesley et al., 2001). Insect genes expression can be downregulated by dsRNA injection (Bettencourt et al., 2002; Eleftherianos et al., 2006; Ohnishi et al., 2006) or with artificial diets containing high concentrations of dsRNA (Turner et al., 2006), but an efficient method of delivering dsRNA to control pests in the field remains to be developed. Some plant-mediated herbivorous insect RNAis have been reported to suppress critical insect genes by feeding insects plant tissues engineered to produce a specific dsRNA (Mao et al., 2007). For example, when larvae are fed plant material expressing dsRNAs specific to *CYP6AE14*, the levels of these transcripts in the midgut are decreased, and larval growth is retarded. These results suggest that feeding insects plant material expressing dsRNA may be a general RNAi strategy and be applicable in entomological and insect pest field control research, which provides us inspiration. According to our study, dsRNA specific to *GSTS1* can be expressed in plants to specifically control *S. litura* damage, but how these successes observed in the laboratory translate into effective pest control in the field remains unknown. However, researchers and farmer can believe that silencing insect-detoxifying genes via plant delivery could be a powerful strategy for controlling insect pests.

In conclusion, while insect-plant interactions have been studied for several years, the mechanisms underlying resistance in insects remain poorly understood, and many key genes and proteins involved in these interactions have not been elucidated. In the present study, we utilized an RNA-Seq approach to investigate control *S. litura* and *S. litura* treated with TOM. In total, 134 and 666 upregulated genes were identified in the *S. litura* midgut and fat body tissues, respectively, among which 30 genes were commonly differentially expressed. In addition, *GSTS1* gene expression was induced by TOM treatment. Our study initially clarified the molecular mechanism underlying the adaptation of *S. litura* to TOM, laying the foundation for subsequent pest control by plant-mediated herbivorous insect RNAi.

## Data Availability Statement

The raw data of the RNA-Seq have been submitted to NCBI Sequence Read Archive (SRA) under BioProject accession PRJNA509528.

## Author Contributions

ZS conceived and designed the experiments. QL, ZS, CX, QS, HW, and RW performed the experiments. QL and ZS performed analysis of the data. QL and ZS wrote the manuscript. ZS, YS, and RZ edited the manuscript.

## Conflict of Interest Statement

The authors declare that the research was conducted in the absence of any commercial or financial relationships that could be construed as a potential conflict of interest.

## Footnotes

1 http://www.ncbi.nlm.nih.gov/

## Supplementary Material

FIGURE S1 Chemical structures of tomatine.

FIGURE S2 GO classification diagram.

FIGURE S3 KOG classification diagram.

TABLE S1 Specific primers used in qRT-PCR.

TABLE S2 Fold change of up-regulated genes in RNA-seq and the qRT-PCR.

TABLE S3 Specific primers used in Synthetic dsRNA.

TABLE S4 Splicing length and frequency distribution of transcripts and unigenes.

TABLE S5 Success rate of gene annotation.
