# Supplementary figures and images for: RNA-Seq Analyses of Midgut and Fat Body Tissues Reveal the Molecular Mechanism Underlying Spodoptera litura Resistance to Tomatine
Source: Front Physiol. 2019 Jan 22;10:8. doi: 10.3389/fphys.2019.00008 (PMC6349761; doi:10.3389/fphys.2019.00008)

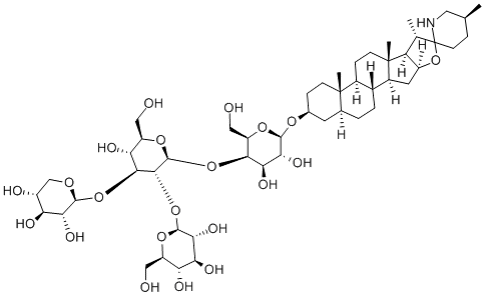

Supplement: FIGURE S1 — Chemical structures of tomatine. [file Image_1.TIF]

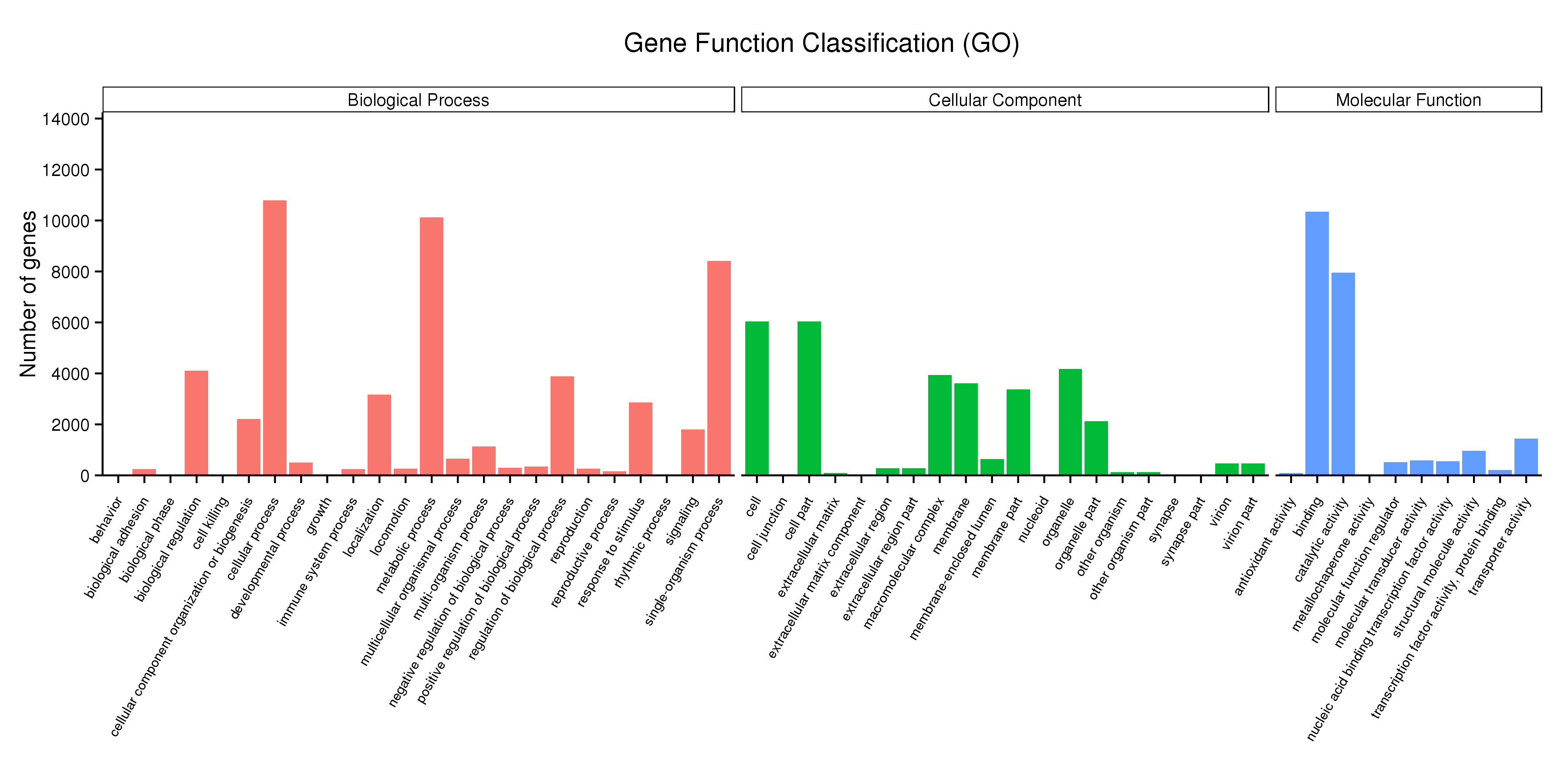

Supplement: FIGURE S2 — GO classification diagram. [file Image_2.PNG]

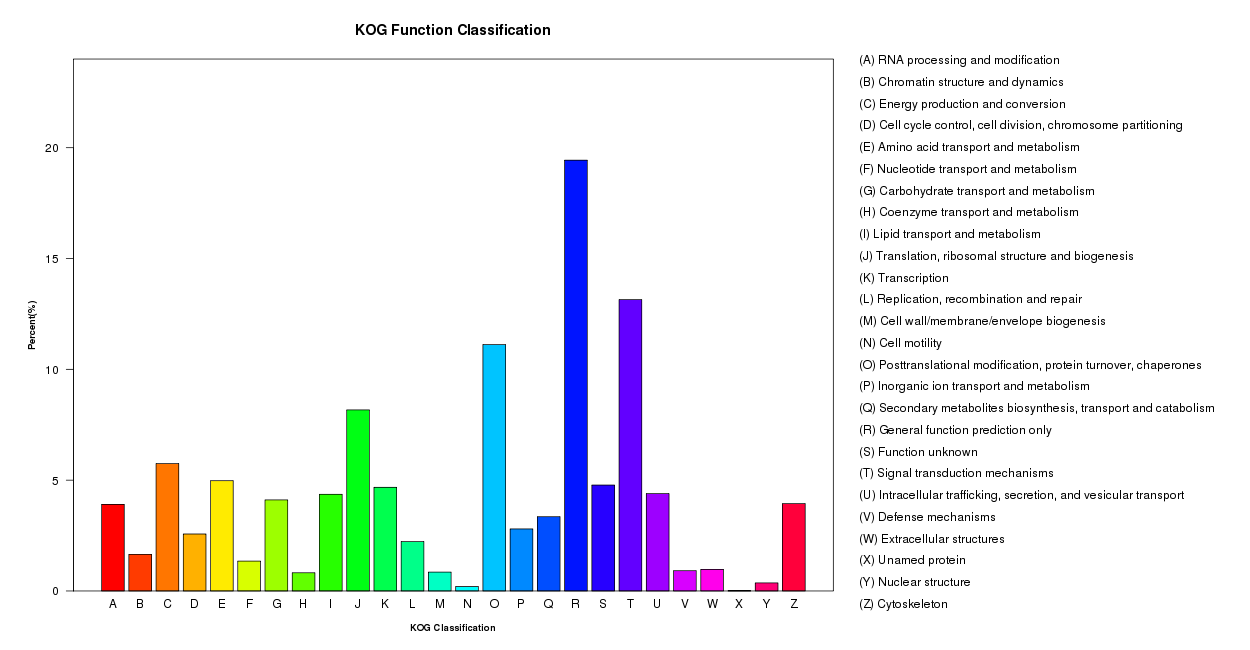

Supplement: FIGURE S3 — KOG classification diagram. [file Image_3.PNG]
