# Supplementary material for: RNA-Seq Analyses of Midgut and Fat Body Tissues Reveal the Molecular Mechanism Underlying Spodoptera litura Resistance to Tomatine
Source: Front Physiol. 2019 Jan 22;10:8. doi: 10.3389/fphys.2019.00008 (PMC6349761; doi:10.3389/fphys.2019.00008)
Supplement: TABLE S1 — Specific primers used in qRT-PCR. [file Table_1.DOCX]

Supplementary Table 1. Specific primers used in qRT-PCR

| Gene Name | Sequence (5’- 3’) |
| --- | --- |
| *CYP4L10*-F | TGGCATTCAGCGCTGGTCCT |
| *CYP4L10*-R | ACTGGGGCGTTACATCAACAGGT |
| *CYP6B6*-F | TGGTGCAGCGTGAAGCTCGT |
| *CYP6B6*-R | ACTGGTAGCAGCGCAAGCGT |
| *ABCG1*-F | ACTGGCCAGCCAATGCAGACA |
| *ABCG1*-R | ATAGCGGCCCCGATGAGCAA |
| *ABCC4*-F | TCGCCGCGTTAGCTACGGTT |
| *ABCC4*-R | ACGCTGTCGATGGCTCTGCT |
| *ABCG4*-F | TCCGCCGCTCTGTTGAGCAT |
| *ABCG4*-R | TAGATAGGGCAGGAGCTGCGGT |
| *UGT33T2*-F | ACGCCGTCCATCAGCCATCAA |
| *UGT33T2*-R | AAGGCGGGGTCTGCTGTGAT |
| *UGT40U1*-F | ACGTGATCAACACACGCGGC |
| *UGT40U1*-R | ACCGCGGCGAGGTCCAAATA |
| *UGT40Q1*-F | AGTCCTCCTCTCAGGCGGTT |
| *UGT40Q1*-R | ATAACGTGGGGTCGCGGTCA |
| *UGT2B18*-F | ACCCTTGTGTCGAGGGCTGA |
| *UGT2B18*-R | TTCGTGTGGAACCTGGCGCT |
| *UG4E*-F | TCCAACGCGCGGAACAGATCA |
| *UG4E*-R | TCAGCGCCGCGAAGTGGATT |
| *CCE016a*-F | TGGTGGGGTCAGCGTCACAT |
| *CCE016a*-R | ACGCTTGTGCCCAGTACGAGA |
| *CCE025a*-F | ACCCGTGCCATTCAAGTGGG |
| *CCE025a*-R | TCGGGGCGCCAGATCTTTCT |
| *CYP4G75*-F | GCAAGCTGATTGCCCCCACT |
| *CYP4G75*-R | AACGGCCCTGGAGTTGGCAT |
| *CYP324A6*-F | ATATTCTGGCGCCGTGGCCT |
| *CYP324A6*-R | TCGGGTCGGACTTGCCAGAA |
| *CYP340AB1*-F | ACAGGTGCTGTTGGCACGCA |
| *CYP340AB1*-R | GTGCACACTGCTGTCCCGTT |
| *CYP4S9v1*-F | TCTGAAAGGGCGCGTCGTCA |
| *CYP4S9v1*-R | TGCGACAGAAGTCCAAAGGTCG |
| *CYP339A1*-F | GCCACCCATGTGTTTGCTCACC |
| *CYP339A1*-R | CGGCGGAACGTTTACCGCTT |
| *CCE006a*-F | ACGGCGGAGCGAGGAACATT |
| *CCE006a*-R | TCATCTGCATGCGCTGCTCCT |
| *ABCF4*-F | TGTGGAGGCCCCACCATCAT |
| *ABCF4*-R | AGCGGCTGCAGCGTCATCTA |
| *ABCA2*-F | TGGAGCCGTAGCCGACGTTA |
| *ABCA2*-R | TGCTTCACGCTGGCCCTCAT |
| *ABCB6*-F | GCCTGCAAAACTGACGCGGT |
| *ABCB6*-R | CAGCGTTCGACACGCCATCT |
| *UGT42C1*-F | ACTTCACGTACGTTTGGCGGGT |
| *UGT42C1*-R | TGTGCCGCCACTTGGTGACT |
| *UGT33J2*-F | TCCGTTCCCCTGTCGCCAAT |
| *UGT33J2*-R | GCAACGGCTAAAGCGGCCAA |
| *UGT33F4*-F | TTGTTCGTGCCACAGGCGGT |
| *UGT33F4*-R | AGAGGCCACGACGTGACAGT |
| *UGT33B13*-F | TGCACGACCAGCCTCAACCT |
| *UGT33B13*-R | TCCGTCCACGACATGTTGGCT |
| *GSTS3*-F | TGCGCTAACGACCGACTACC |
| *GSTS3*-R | ACTCTTGGTACACGTGGGCT |
| *GSTE11*-F | TCCTGGCGGCCGTTCCATAA |
| *GSTE11*-R | TAAGCAGCTGCGCGGAATCG |
| *GSTE2*-F | TGTTGGTGCCACCGCCATCT |
| *GSTE2-*R | TTGTCCATCCACGCAGCCGT |
| *GSTE13*-F | GCCCTGTACCCCAAATTATCGGC |
| *GSTE13*-R | AATTCTTGCAGACCAGGCAAGT |
| *GSTS2*-F | TAAGAACAACGGCCACATCGC |
| *GSTS2*-R | CCTTCTGGAATGCTGGGTATTTCT |
| *GSTS1*-F | TGGCAAGCTTTCCTGGGGTGA |
| *GSTS1*-R | TGCACCAAAGTACCAACAGCTGGA |
| *GSTZ2*-F | TTGCGTCGGAGACCAGCTCA |
| *GSTZ*2-R | ACTGATGTCCAGCGCGTGTCT |
| *GSTS5*-F | TGCCTGACATCCTCAAATTCTTGCT |
| *GSTS5*-R | TGCTCAGTACGCTCTAATCAGTCG |
| *TR*-F | TCGCCACGTTCGTCTGCTCA |
| *TR*-R | ATTGCTCGCCACCGGACGAT |
| *GS*-F | AGCTCCATGCCTTCGTGCCA |
| *GS*-R | GGCCAGGAAATGGGACCTTCGT |
| SL-EF-1α-F | ACAGACAAGCCCCTGCGTCT |
| SL-EF-1α-R | TTGTCACCGGGTACGGCCTCTT |
