# Supplementary material for: RNA-Seq Analyses of Midgut and Fat Body Tissues Reveal the Molecular Mechanism Underlying Spodoptera litura Resistance to Tomatine
Source: Front Physiol. 2019 Jan 22;10:8. doi: 10.3389/fphys.2019.00008 (PMC6349761; doi:10.3389/fphys.2019.00008)
Supplement: TABLE S2 — Fold change of up-regulated genes in RNA-seq and the qRT-PCR. [file Table_2.DOCX]

Supplementary Table 2. Fold change of up-regulated genes in RNA-seq and the qRT-PCR

| Gene | Fold change (RNA-seq) | q.value | Fold change (qRT-PCR) | Tissue |
| --- | --- | --- | --- | --- |
| CYP4L10 | 2.14 | 5.03E-17 | 1.51 | M |
| CYP6B6 | 2.90 | 5.75E-142 | 3.82 | M |
| ABCG1 | 10.33 | 1.17E-18 | 9.40 | M |
| ABCC4 | 4.76 | 2.02E-06 | 9.19 | M |
| ABCG4 | 3.36 | 5.51E-13 | 3.87 | M |
| UGT33T2 | 4.95 | 1.15E-06 | 3.17 | M |
| UGT40U1 | 1.89 | 1.85E-04 | 2.43 | M |
| **UGT40Q1** | **1.77** | **3.00E-05** | **0.65** | **M** |
| UG4E | 1.59 | 5.71E-05 | 1.30 | M |
| CCE016a | 3.14 | 2.52E-21 | 2.75 | M |
| CCE025a | 2.30 | 2.35E-06 | 1.94 | M |
| CYP4G75 | 1.79 | 1.33E-09 | 2.32 | F |
| CYP324A6 | 3.89 | 5.93E-47 | 4.68 | F |
| CYP340AB1 | 3.90 | 2.24E-04 | 3.32 | F |
| CYP4S9v1 | 1.68 | 1.28E-08 | 1.72 | F |
| CYP339A1 | 1.68 | 3.20E-06 | 1.72 | F |
| CCE006a | 4.87 | 1.11E-125 | 1.25 | F |
| ABCF4 | 2.27 | 4.64E-03 | 2.64 | F |
| ABCA2 | 2.39 | 4.97E-03 | 2.52 | F |
| ABCB6 | 1.53 | 2.89E-04 | 2.35 | F |
| UGT42C1 | 6.32 | 5.54E-07 | 5.60 | F |
| UGT33J2 | 5.49 | 2.91E-20 | 4.66 | F |
| UGT33F4 | 22.35 | 5.54E-17 | 18.99 | F |
| UGT33B13 | 3.73 | 7.50E-05 | 1.73 | F |
| GSTS3 | 5.51 | 3.95E-04 | 2.51 | F |
| GSTE11 | 2.24 | 1.90E-05 | 1.82 | F |
| GSTE2 | 1.90 | 4.26E-22 | 1.96 | F |
| GSTE13 | 1.68 | 3.35E-03 | 1.30 | F |
| GSTS2 | 1.89 | 5.68E-15 | 2.04 | F |
| GSTS1 | 8.36 | 3.75E-10 | 6.77 | F |
| GSTZ2 | 1.79 | 3.42E-06 | 2.33 | F |
| GSTS5 | 3.01 | 7.04E-61 | 2.48 | F |
| TR | 1.77 | 4.35E-08 | 1.75 | F |
| GS | 2.33 | 4.59E-03 | 3.34 | F |

Fold change represents the scale of the expression levels of TOM treatment/Control.

M: Midgut; F: Fat body
