# Supplementary material for: RNA-Seq Analyses of Midgut and Fat Body Tissues Reveal the Molecular Mechanism Underlying Spodoptera litura Resistance to Tomatine
Source: Front Physiol. 2019 Jan 22;10:8. doi: 10.3389/fphys.2019.00008 (PMC6349761; doi:10.3389/fphys.2019.00008)
Supplement: TABLE S3 — Specific primers used in Synthetic dsRNA. [file Table_3.DOCX]

Supplementary Table 3. Specific primers used in Synthetic dsRNA

| Gene Name | Sequence (5’- 3’) |
| --- | --- |
| T7-GFP-dsRNA-F | GGATCCTAATACGACTCACTATAGGAAGGGCGAGGAGCTGTTCACCG |
| T7-GFP-dsRNA-R | GGATCCTAATACGACTCACTATAGGCAGCAGGACCATGTGATCGCGC |
| GFP-dsRNA-F | AAGGGCGAGGAGCTGTTCACCG |
| GFP-dsRNA-R | CAGCAGGACCATGTGATCGCGC |
| T7-*GSTS1*-dsRNA-F | GGATCCTAATACGACTCACTATAGGGTCTTGCCGAACCCATAA |
| T7-*GSTS1*-dsRNA-R | GGATCCTAATACGACTCACTATAGGTAGCCTTGACATACTCCTTCACT |
| *GSTS1*-dsRNA-F | GTCTTGCCGAACCCATAA |
| *GSTS1*-dsRNA-R | TAGCCTTGACATACTCCTTCACT |
