# Supplementary material for: RNA-Seq Analyses of Midgut and Fat Body Tissues Reveal the Molecular Mechanism Underlying Spodoptera litura Resistance to Tomatine
Source: Front Physiol. 2019 Jan 22;10:8. doi: 10.3389/fphys.2019.00008 (PMC6349761; doi:10.3389/fphys.2019.00008)
Supplement: TABLE S4 — Splicing length and frequency distribution of transcripts and unigenes. [file Table_4.DOCX]

Supplementary Table 4. Splicing length and frequency distribution of transcripts and unigenes
